# Ultrasound-Assisted Synthesis of 4-Alkoxy-2-methylquinolines: An Efficient Method toward Antitubercular Drug Candidates

**DOI:** 10.3390/molecules26051215

**Published:** 2021-02-24

**Authors:** Ana Flávia Borsoi, Josiane Delgado Paz, Kenia Pissinate, Raoní Scheibler Rambo, Víctor Zajaczkowski Pestana, Cristiano Valim Bizarro, Luiz Augusto Basso, Pablo Machado

**Affiliations:** 1 Centro de Pesquisas em Biologia Molecular e Funcional, Instituto Nacional de Ciência e Tecnologia em Tuberculose, Pontifícia Universidade Católica do Rio Grande do Sul, 90616-900 Porto Alegre, Rio Grande do Sul, Brazil; afborsoi@gmail.com (A.F.B.); josiane.delgado@acad.pucrs.br (J.D.P.); k.pissinate@gmail.com (K.P.); raoni.rambo@gmail.com (R.S.R.); victorzaj@hotmail.com (V.Z.P.); cristiano.bizarro@pucrs.br (C.V.B.); luiz.basso@pucrs.br (L.A.B.); 2 Programa de Pós-Graduação em Medicina e Ciências da Saúde, Pontifícia Universidade Católica do Rio Grande do Sul, 90616-900 Porto Alegre, Rio Grande do Sul, Brazil; 3 Programa de Pós-Graduação em Biologia Celular e Molecular, Pontifícia Universidade Católica do Rio Grande do Sul, 90616-900 Porto Alegre, Rio Grande do Sul, Brazil

**Keywords:** sonochemistry, heterocyclic synthesis, new methods, antituberculosis drug discovery

## Abstract

Tuberculosis (TB) has been described as a global health crisis since the second half of the 1990s. *Mycobacterium tuberculosis* (Mtb), the etiologic agent of TB in humans, is a very successful pathogen, being the main cause of death in the population among infectious agents. In 2019, it was estimated that around 10 million individuals were contaminated by this bacillus and about 1.2 million succumbed to the disease. In recent years, our research group has reported the design and synthesis of quinoline derivatives as drug candidates for the treatment of TB. These compounds have demonstrated potent and selective growth inhibition of drug-susceptible and drug-resistant Mtb strains. Herein, a new synthetic approach was established providing efficient and rapid access (15 min) to a series of 4-alkoxy-6-methoxy-2-methylquinolines using ultrasound energy. The new synthetic protocol provides a simple procedure utilizing an open vessel system that affords the target products at satisfactory yields (45–84%) and elevated purities (≥95%). The methodology allows the evaluation of a larger number of molecules in assays against the bacillus, facilitating the determination of the structure–activity relationship with a reduced environmental cost.

## 1. Introduction

Despite the advances achieved in the last decade, tuberculosis (TB) remains a serious public health problem. According to the World Health Organization, 10 million new cases and 1.2 million deaths were reported worldwide in 2019 [1]. Caused mainly by *Mycobacterium tuberculosis* (Mtb), the disease has been described as the main cause of death by a single infectious agent [1]. One of the main difficulties for the treatment of TB has been the emergence of strains resistant to clinically available drugs [2]. Infections caused by Mtb strains resistant to rifampicin (RR-TB), resistant to rifampicin and isoniazid (MDR-TB), or even resistant to rifampicin and isoniazid in addition to any fluoroquinolone and injectable second-line drugs (XDR-TB) have created problems for health systems globally. After a long period without new alternatives on the market, the last decade has witnessed the approval of three drugs. “Bedaquiline (2012) [3], delamanid (2014) [4], and pretomanid (2019) [5]” are new approved drugs for the treatment of TB caused by resistant strains. However, the adaptive capacity of mycobacteria with the possibility of rapid resistant strain emergence [6] demonstrates that new drug candidates for the treatment of TB should still be a high priority on the global health agenda.

In this context, our research group has reported the design and synthesis of 2-(quinolin-4-yloxy)acetamides as drug candidates for the treatment of TB with some encouraging results [7,8,9]. Recently, using a molecular simplification strategy, a series of 2-(quinolin-4-yloxy)acetamide-based compounds was synthesized [9]. These molecules exhibited potent and selective growth inhibition of drug-susceptible and drug-resistant Mtb strains with minimal inhibitory concentrations (MICs) in the submicromolar range. In addition, the synthesized structures that carry out their antitubercular activity by targeting the cytochrome bc1 complex showed improved metabolic properties compared with their counterparts and were able to inhibit the intracellular growth of Mtb [9].

As part of our research program, we are interested in the development of alternative and environmentally benign methodologies to obtain pharmacologically active compounds. The possibility of aligning green chemistry concepts with obtaining drug candidates has been of great interest in both the pharmaceutical sector and scientific community [10,11]. The pharmaceutical industry has the highest unit cost of production and generates the largest amount of waste among the chemical processing industries [12]. These costs of production have been related, among other factors, to the efficiency of the processes used to obtain products with high purity, which allows their pharmaceutical use; the numbers and time spent on the unit operations; the amount of solvent with a subsequent need for treatment; and the energy spent on the process. Within this context, developing more efficient methodologies with lower environmental costs in the early drug discovery stages has been highly recommendable [10,11]. As an alternative to classical methods, sonochemical-based protocols have been used to increase the rates of a wide variety of reactions, resulting in products generated under milder conditions with a significant reduction in reaction times [13,14]. These methods have been described as more environmentally friendly and sustainable and are associated with greater selectivity and lower energy consumption for the desired transformations [15]. The mechanism of such methods is based on the phenomenon of acoustic cavitation, which induces unique conditions of pressure and temperature through the formation, growth, and adiabatic collapse of bubbles in the liquid medium [13,14]. This effect improves mass transfer and increases turbulent flow in the liquid, facilitating the chemical transformations. In our studies, the use of ultrasound has led to the production of compounds in reduced reaction times with high yields and purity [16,17]. Such characteristics have increased the number of compounds evaluated in pharmacological models, contributing to accelerating the hit to lead optimization process.

Therefore, because of the interesting antitubercular activity of 2-(quinolin-4-yloxy)acetamide-based compounds [9], in an attempt to improve the synthetic protocol, a new ultrasound-assisted method for obtaining these compounds is described.

## 2. Results

Regarding ultrasound-based methods for *O*-alkylation of heterocyclic compounds, existing protocols have described the treatment of 3-hydroxychromones [18] or 5-hydroxychromone [19] with alkyl(allyl) halides in the presence of potassium carbonate (K_2_CO_3_) as the base for the synthesis of the desired products. In these procedures, dimethylformamide (DMF) and *N*-methylpyrrolidinone were used as solvents to furnish the compounds with satisfactory yields.

Thus, ultrasound-assisted *O*-alkylation reactions of 6-methoxy-2-methylquinolin-4-ol (**1**) were performed in the presence of K_2_CO_3_ as the base and DMF as the solvent. Nucleophilic substitution reaction was achieved by using appropriate reagents and reactants in an open vessel system after sonication for 15 min (Table 1). When reduced reaction times were tested, the product **3a** was isolated with lower yields or with incomplete conversion based on high-performance liquid chromatography (HPLC) analysis. In addition, the 1:1.2:3 molar ratio between quinoline **1a**, benzyl bromide (**2a**), and K_2_CO_3_, respectively, provided higher yields when compared with other concentrations (Table 1, Entries 5 and 6). It is noteworthy that the absence of K_2_CO_3_ significantly reduced the conversion to compound **2a** (Table 1, Entry 7). Finally, the modification of solvents did not produce better results when compared with the reactions performed in DMF. In order to verify the existence of a catalytic effect produced by ultrasonic waves on the synthetic process under study, the synthesis of compound **3a** was performed under conventional thermal heating using the same experimental conditions described in the ultrasound-assisted procedure. After 15 min at 120 °C, the reaction provided a mixture of **1a**:**3a** at a ratio of 17:83, respectively, based on HPLC determination.

Afterwards, the protocol was extended to other benzyl bromides (**2**), leading to compounds **3a**–**n** with 45–76% yields (Table 2). Spectroscopic and spectrometric data were obtained to agree with the proposed structures already described [9]. Additionally, the possibility of obtaining *N*-alkylated products, using this method followed by aqueous work-up, has been excluded based on data already described in the literature [7]. Interestingly, compounds purities were >95% based on HPLC determinations. This finding allows the use of such compounds in pharmacological assays without the need for any further purification procedure. Additionally, electronic variations of the substituents did not significantly alter the observed results. In addition, the methods described in the literature have reported the synthesis of compounds from quinoline and benzyl bromide derivatives in the presence of K_2_CO_3_ in DMF under conventional conditions with reaction times ranging from 5 to 8 h [20,21]. These procedures have led to products with 17–76% yields, one of which described the use of chromatography in the purification of compounds. In particular, compounds **3a**–**n** have been synthesized under conventional conditions in the presence of K_2_CO_3_ in DMF [9]. The products were obtained after stirring for 18 h at 25 °C with 49–95% yields (Table 2). Comparing the conventional method with sonochemical conditions, the ultrasound protocol highly reduced the reaction time for the *O*-alkylation of quinoline derivative compounds. In addition, the method provided the compounds at high purity without the need for any additional purification procedures. Synthesized structures were endowed with potent antimycobacterial activity, with MICs in the range of 0.3 to 30.8 µM [9].

In summary, the preparation of quinoline derivative compounds has been successfully accomplished via sonochemical conditions. The simplicity of execution, significantly shorter reaction times (15 min), satisfactory yields (45–84%), and the purity of the isolated products make this protocol attractive. The use of the ultrasound-assisted method described has been applied in the generation of a wide variety of compounds with potent antimycobacterial activity (MIC = 0.3–30.8 µM [9]) through a method that has lower environmental and operational costs compared with existing ones.

## 3. Experimental

### 3.1. Chemistry

Commercially available reactants and solvents were obtained from commercial suppliers, and were used without additional purification. The reactions were carried out with a standard probe (25 mm) connected to a 1500 Watt Sonics Vibra-Cell ultrasonic processor (Newtown, CT, USA) equipped with integrated temperature control. The device operates at 20 kHz, and the amplitude was set to 20% of the maximum power output. In all reactions, the temperature was raised to 115–120 °C after sonication for 7–8 min. The progress of the reaction was monitored using thin-layer chromatography (TLC, Kenilworth, NJ, USA) with Merck TLC Silica gel 60 F254. The melting points were measured using a Microquímica MQAPF-302 apparatus. ^1^H and ^13^C NMR spectra were acquired on an Avance III HD Bruker spectrometer (Bruker Corporation, Fällanden, Switzerland). Chemical shifts (*δ*) were expressed in parts per million (ppm) relative to DMSO-*d*_6_, which was used as the solvent, and to TMS, which was used as an internal standard. High-resolution mass spectra (HRMS) were obtained for all compounds on an LTQ Orbitrap Discovery mass spectrometer (Thermo Fisher Scientific, Bremen, Germany). The analyses were performed through the direct infusion of the sample in MeOH/MeCN (1:1) with 0.1% formic acid (flow rate 10 mL/min), in a positive-ion or negative-ion mode using electrospray ionization (ESI). For elemental composition, calculations were performed using the specific tool included in the Qual Browser module of Xcalibur software. The compound purity was determined using an Äkta HPLC system (GE Healthcare^®^ Life Sciences) equipped with a binary pump, manual injector, and UV detector. Unicorn 5.31 software (Build 743) was used for data acquisition and processing. The HPLC conditions were as follows: RP column, 5 µm Nucleodur C-18 (250 × 4.6 mm); flow rate, 1.5 mL/min; UV detection, 254 nm; 100% water (0.1% acetic acid) was maintained from 0 to 7 min, followed by a linear gradient from 100% water (0.1% acetic acid) to 90% acetonitrile/methanol (1:1, *v/v*) from 7 to 15 min (15–30 min) and subsequently returned to 100% water (0.1% acetic acid) in 5 min (30–35 min) and maintained for an additional 10 min (35–45 min).

### 3.2. General Procedure for the Synthesis of 6-Methoxy-2-Methylquinolines

6-Methoxy-2-methylquinolin-4-ol (**1**) (1 mmol), benzyl bromides (**2**) (1.2 mmol), and K_2_CO_3_ (0.415 g, 3 mmol) were mixed in DMF (20 mL) in a 100 mL beaker. The reaction mixture was sonicated for 15 min using an ultrasonic probe. Afterwards, the amount of solvent was decreased under reduced pressure, and the resultant mixture was diluted in water. Subsequently, the solid formed was filtered off and washed under agitation with water (3 × 20 mL); after each wash, the product was separated from the liquid phase by centrifugation (10 min, 18,000 rpm at 4 °C). Finally, the product was dried under reduced pressure for at least 24 h. Analytical data were found to be in agreement with the already reported data [9]. Compounds **3a** and **3b** were selected for exemplification. 

4-(Benzyloxy)-6-methoxy-2-methylquinoline (**3a**): yield 84%; white to slightly yellow solid; m.p.: 140–141 °C; HPLC 95% (*t*_R_ = 14.57 min); ^1^H NMR (400 MHz, DMSO-*d*_6_) *δ* 2.55 (s, 3H, CH_3_), 3.84 (s, 3H, CH_3_), 5.38 (s, 2H, CH_2_), 7.00 (s, 1H, Qu-H*), 7.40–7.30 (m, 3H, Qu-H*, Ph-H), 7.49–7.41 (m, 2H, Ph-H), 7.58 (dt, *J* = 6.3, 1.4 Hz, 2H, Qu-H*, Ph-H), 7.79 (d, *J* = 9.1 Hz, 1H, Qu-H*); HRMS (FTMS + pESI) *m/z* calcd. for C_18_H_17_NO_2_ (M)^+^: 280.1332; found: 280.1325; Qu-H*: quinolines hydrogens.

4-((4-Fluorobenzyl)oxy)-6-methoxy-2-methylquinoline (**3b**): yield 62%; white solid; mp.: 114–115 °C; HPLC 95% (*t*_R_ = 15.12 min); ^1^H NMR (400 MHz, DMSO-*d*_6_) *δ* 2.59 (s, 3H, CH_3_), 3.84 (s, 3H, CH_3_), 5.35 (s, 2H, CH_2_), 6.98 (s, 1H, Qu-H*), 7.40–7.25 (m, 4H, Ph-H, Qu-H*), 7.69–7.58 (m, 2H, Ph-H, Qu-H*), 7.81 (d, *J* = 9.0 Hz, 1H, Qu-H*); HRMS (FTMS + pESI) *m/z* calcd. for C_18_H_16_FNO_2_ (M)^+^: 298.1238; found: 298.1245; Qu-H*: quinolines hydrogens. 

## Figures and Tables

**Table 1:**
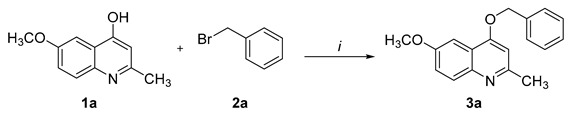
Reagents and conditions: *i* = K_2_CO_3_, solvent, ultrasound or conventional, 5–20 min. ^a^ Isolated yields. ^b^ Reaction performed under conventional thermal heating (120 °C).

Entry	Time (min)	Molar Ratio (1a:2a:K_2_CO_3_)	Solvent	Yield (%) ^a^ or (Ratio 1a:3a)
1	5	1:1.2:3	DMF	(21:79)
2	10	1:1.2:3	DMF	65
3	15	1:1.2:3	DMF	84
4	20	1:1.2:3	DMF	81
5	15	1:1.2:2	DMF	59
6	15	1:1.2:1.2	DMF	48
7	15	1:1.2:0	DMF	(99:1)
8	15	1:1.2:3	CH_3_CN	73
9	15	1:1.2:3	EtOH	44
10	15	1:1.2:3	DMF	(17:82) ^b^

**Table 2:**
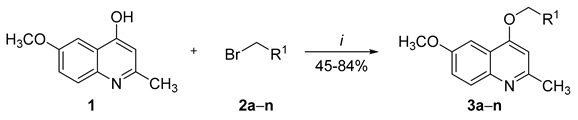
Conditions: *i* = K_2_CO_3_, DMF, 15 min. ^a^ Isolated yields. ^b^ Ultrasound-assisted method. ^c^ Conventional stirring method [9].

Entry	R^1^	Yield (%) ^a,b^	Yield (%) ^a,c^
**a**	Ph	84	49
**b**	F-4-C6H4	62	67
**c**	F-2-C6H4	45	52
**d**	(F)2-3,4-C6H3	83	90
**e**	Cl-4-C6H4	72	73
**f**	Cl-3-C6H4	79	90
**g**	Cl-2-C6H4	74	86
**h**	(Cl)2-3,4-C6H3	73	63
**i**	Br-3-C6H4	76	95
**j**	F3C-4-C6H4	81	81
**k**	F3C-3-C6H4	82	90
**l**	O2N-4-C6H4	50	64
**m**	iPr-4-C6H4	68	80
**n**	2-Naphthyl	57	64

## Sample Availability

Samples of the synthesized compounds are available from the authors.
